# Death of Dr. Wooten

**Published:** 1906-09

**Authors:** 


Death of Dr. Wooten.—Dr. Thomas D. Wooten, of Austin, Texas, died at Eureka Springs, Ark., July 31, 1906, aged 77 years. Dr. Wooten was a "landmark" in Texas. Jointly with the late Dr. Ashbel Smith, he may be said to have been the father of the Medical Department of the University of Texas, although he never held a chair in that institution. For some years he was, however, President of the Board of Regents of The University of Texas, and always one of the pillars of that great school. Dr. Wooten was a distinguished Confederate surgeon, he having been Chief Surgeon of Division in Price's Army in Missouri. His reputation as a surgeon was not confined to Texas, but extended throughout the South. Dr. Wooten was a Kentuckian; born in Barren county, Ky., March 6, 1829. His wife was a Goodall—a noted family of Kentucky; and he leaves two sons—eminent physicians, on whom the mantle of his reputation falls,—to perpetuate the name and fame of their distinguished sire.

